# Hybrid repair of Kommerell's diverticulum with aberrant subclavian artery

**DOI:** 10.1016/j.jvscit.2026.102129

**Published:** 2026-01-05

**Authors:** Christian H. Summa, Christopher A. DeMaioribus, Daniel Swink, Joseph AbouAyash, Shivprasad Nikam, Boyoung Song, Evan J. Ryer, Melissa A. Obmann

**Affiliations:** a Department of Endovascular and Vascular Surgery, Geisinger Wyoming Valley Medical Center, Wilkes Barre, PA; b Department of Endovascular and Vascular Surgery, Geisinger Medical Center, Danville, PA; c Geisinger Commonwealth School of Medicine, Scranton, PA

**Keywords:** Kommerell's diverticulum, Aberrant subclavian artery, Hybrid repair, Thoracic endovascular aortic repair (TEVAR), Carotid-subclavian bypass, Endoleak

## Abstract

Kommerell's diverticulum (KD) is a rare aortic arch anomaly associated with aberrant subclavian arteries and risk of rupture. We present an 80-year-old woman with a right-sided aortic arch and aberrant left subclavian artery arising from a 5.0-cm KD. A staged hybrid repair was performed: right carotid-subclavian bypass, thoracic endovascular aortic repair, and embolization of the aberrant subclavian artery. Postoperative imaging confirmed complete exclusion of the diverticulum and a patent bypass graft. This case illustrates the safety and effectiveness of hybrid repair for KD in complex arch anatomy, offering a less invasive alternative to open surgery.

Kommerell's diverticulum (KD) is a rare congenital aortic arch anomaly characterized by an aneurysmal dilatation at the origin of an aberrant subclavian artery.^1^ Patients with KD may be asymptomatic or present with compressive symptoms caused by the aberrant artery's course behind mediastinal structures. Common presentations include dysphagia lusoria (esophageal compression) and tracheal compression leading to stridor or cough.^2^ Although KD is an anatomical variant, its presence is associated with a significant risk of aortic complications, with reported rates of rupture or dissection as high as 19% to 53% if left untreated.^2^ No single standard surgical strategy for KD has been established, although multiple open and endovascular techniques have been described.^3^ We present a case of a large KD in an elderly patient that was managed successfully with a staged hybrid approach (carotid-subclavian bypass and thoracic endovascular stent grafting).

## Case report

**Fig 1:**
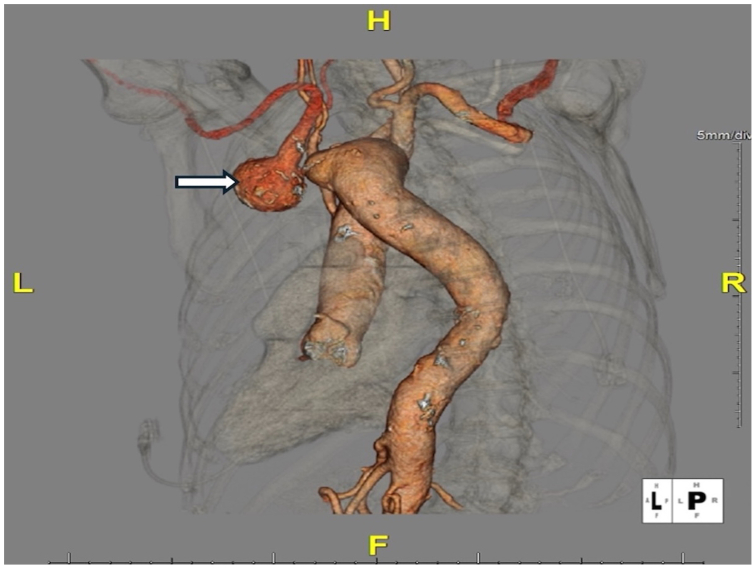
Preoperative three-dimensional reconstruction of chest computed tomography angiography (CTA) showing a right-sided aortic arch with an aberrant left subclavian artery arising from a Kommerell diverticulum (*white arrow*). The saccular aneurysmal dilation at the origin of the left subclavian artery is visualized posterior to the esophagus.

An 80-year-old woman with a history of hypertension and tobacco use was referred for evaluation of a 4-cm left subclavian artery aneurysm incidentally noted on chest radiography. Computed tomography angiography (CTA) of the chest revealed a right-sided aortic arch with an aberrant left subclavian artery arising from a large diverticulum on the proximal descending thoracic aorta (KD). The arch branching pattern was atypical: the left common carotid artery arose first from the ascending aorta and bifurcated in the upper chest, followed by the right common carotid and right subclavian arteries. The aberrant left subclavian artery was the fourth branch, originating from the diverticulum and coursing retroesophageally. Its retroesophageal course caused a focal stenosis, and just distal to this area was a saccular aneurysmal dilatation measuring approximately 4 cm (Fig 1). Given the aneurysm's size and the potential risk of rupture, surgical repair was recommended despite the patient being asymptomatic. A written consent form was obtained from the patient to report her case details and imaging studies.

We planned a hybrid repair, beginning with supra-aortic debranching to maintain perfusion to the left arm. The first stage was a right common carotid artery to left subclavian artery bypass. The right carotid artery was chosen as the inflow because the patient's left common carotid artery had an atypical intrathoracic origin with moderate stenosis, making it a less ideal source. Through separate supraclavicular incisions, the right common carotid artery and left subclavian artery were exposed. An orogastric tube was placed to facilitate a retropharyngeal tunneling approach. A tunnel was bluntly created in the retropharyngeal space (anterior to the cervical spine, posterior to the esophagus) connecting the two incisions. An 8-mm ringed polytetrafluoroethylene graft (GORE-TEX, W. L. Gore & Associates) was passed through this tunnel and anastomosed end-to-side to the right common carotid and left subclavian artery. The bypass was completed without complication. The patient tolerated the procedure well, and postoperative recovery was uneventful.

Several weeks later, the patient returned for endovascular exclusion of the KD. A thoracic aortic endograft (Cook Alpha Thoracic Endovascular Graft, Cook Medical) was deployed in the descending thoracic aorta by percutaneous access through the right groin, covering the origin of the aberrant left subclavian artery/KD. This maneuver effectively sealed the diverticulum and excluded the aneurysmal segment of the subclavian artery. A postdeployment aortogram showed good graft apposition. However, a small type II endoleak was noted filling the aneurysm sac. The patient experienced no complications and was discharged in stable condition.

**Fig 2:**
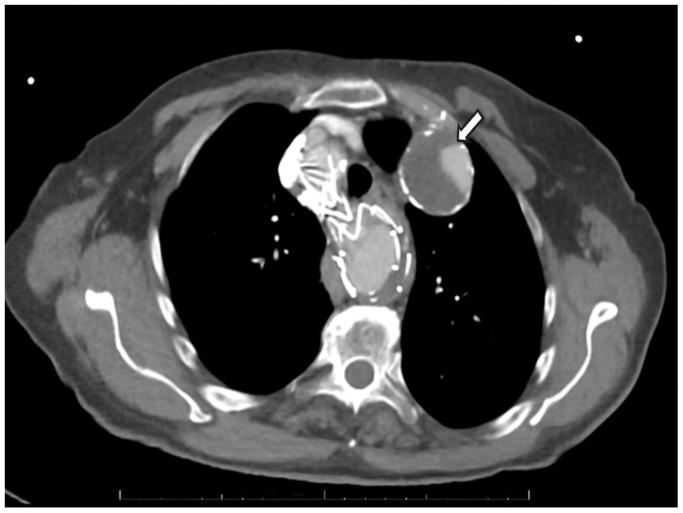
Status post 1 month computed tomography angiography (CTA) imaging in the axial plane demonstrating persistent type II endoleak (*white arrow*) before additional interventions.

**Fig 3:**
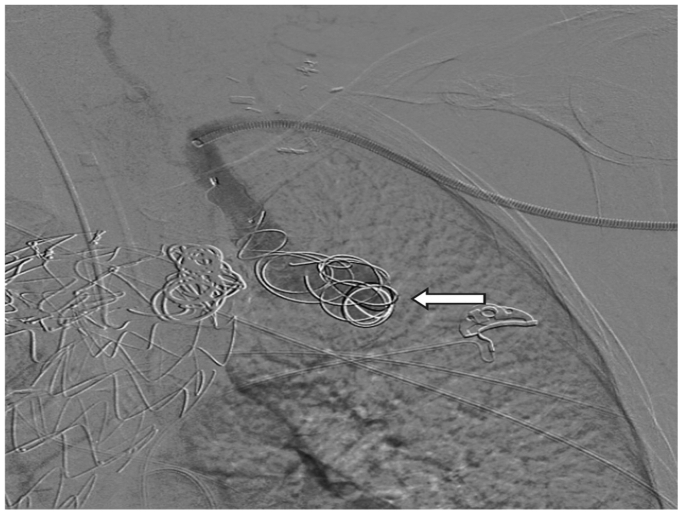
Intraoperative angiogram demonstrating deployment of multiple coils (Terumo Azur CX detachable coils, Terumo) into the aneurysm sac (*white arrow*).

One month later, surveillance CTA demonstrated a persistent type II endoleak (Fig 2). To address the endoleak, a third-stage intervention was undertaken. We had initially planned to treat the endoleak in the prior thoracic endovascular aortic repair (TEVAR) with an occluder plug in the left subclavian artery distal to the subclavian aneurysm by the right groin approach. However, a high-grade stenosis at the origin of the left subclavian artery from the KD did not allow planned intervention during the thoracic endograft placement. Through a small incision in the left arm (above the antecubital fossa), the left brachial artery was accessed, and a catheter was advanced retrograde into the left subclavian artery. Angiography confirmed persistent perfusion of the aneurysm sac via the aberrant subclavian stump. A 6F sheath was positioned, and multiple coils (Terumo Azur CX detachable coils, Terumo Medical) were deployed into the diverticulum and aneurysm sac to promote thrombosis (Fig 3). Additionally, an Amplatzer II vascular plug (Abbott Cardiovascular) was deployed in the proximal left subclavian artery (just before the origin of the left vertebral artery) to prevent any further retrograde flow into the sac. Postembolization angiography showed no filling of the aneurysm. The patient tolerated the procedure well and was discharged the same day.

**Fig 4:**
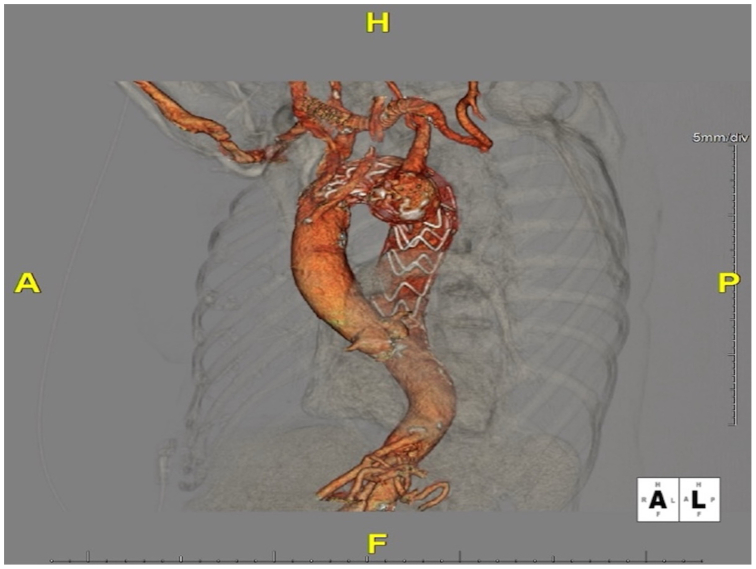
Postoperative three-dimensional computed tomography angiography (CTA) reconstruction after thoracic endovascular aortic repair (TEVAR) and coil embolization, showing successful placement of the thoracic endograft.

Follow-up CTA of the chest several months later demonstrated complete exclusion of the diverticulum without any residual endoleak. The right carotid-left subclavian bypass graft remained patent, and the aneurysm sac had thrombosed with no increase in size (Fig 4). At subsequent clinic visits, the patient continued to do well with intact left arm perfusion and no new symptoms.

## Discussion

KD is a rare congenital vascular anomaly arising at the origin of an aberrant subclavian artery.^4^ The more common configuration involves a left-sided aortic arch with an aberrant right subclavian artery; a right-sided arch with aberrant left subclavian artery, as seen in our case, occurs in only 0.05% of the population.^5^^,^^6^ KD forms owing to incomplete regression of the embryologic aortic arch system, specifically persistence of the right dorsal aorta. Resulting in a dilated segment at the origin of the aberrant artery.^6^ Although many patients are asymptomatic, KD can produce symptoms like dysphagia or dyspnea.^5^ Reported rupture or dissection rates range from 19% to >50%, underscoring the importance of careful surveillance and timely intervention.^6^ There are no universal treatment guidelines, but many authors recommend intervention for symptomatic cases, diverticula ≥3 cm at the orifice, or aneurysms ≥5 cm in total diameter.^4^^,^^7^ Traditional open repair involves thoracotomy or sternotomy with cardiopulmonary bypass and subclavian artery reimplantation. Although effective, such procedures can pose significant morbidity, especially in elderly patients.^5^

Endovascular treatment with thoracic endografts (TEVAR) provides a less invasive alternative, but it may be limited by anatomical constraints. Inadequate landing zones or the need to preserve arch branches can complicate pure endovascular approaches. For this reason, hybrid techniques—combining supra-aortic debranching with TEVAR—have gained traction.^5^^,^^7^ In our case, a three-stage hybrid approach was used: right carotid-to-left subclavian artery bypass, TEVAR with exclusion of the KD, and subsequent coil and plug. The right carotid was selected for inflow owing to unfavorable anatomy and stenosis of the left carotid artery. This approach enabled safe exclusion of the aneurysmal diverticulum without compromising arm or vertebrobasilar perfusion. The staged strategy also allowed for controlled recovery and monitoring between procedures. Our experience aligns with prior series demonstrating the feasibility and safety of hybrid repair for complex arch anatomy and emphasizes the need to take an individualized on an as-needed approach guided by anatomical variations seen in patients with KD.^5^^,^^7^^,^^8^ Successful outcomes hinge on accurate imaging, procedural planning, and timely embolization to address potential type II endoleaks. Although long-term data are limited, intermediate follow-up in our patient shows durable exclusion of the KD, resolution of symptoms, and no endoleak. Continued reporting of such cases will help to refine indications and techniques.

## Conclusions

KD is a rare aortic arch anomaly that can cause compressive symptoms or lead to rupture. Management should be based on symptoms and aneurysm size. This case demonstrates that a staged hybrid approach—carotid-subclavian bypass followed by TEVAR with embolization—is a safe and effective option. Treatment should be individualized to patient anatomy, and continued case reporting will help to refine management strategies.

## Funding

None.

## Disclosures

None.
